# 5-Amino-3-methyl-1-phenyl-1*H*-1,2,4-triazole

**DOI:** 10.1107/S1600536808005801

**Published:** 2008-03-07

**Authors:** Fatma Allouch, Fatma Zouari, Fakher Chabchoub, Mansour Salem

**Affiliations:** a Laboratoire de Chimie Appliquée: Hétérocycles, Corps gras et Polymères, Faculté des Sciences de Sfax, BP 802, 3018 Sfax, Tunisia; b Laboratoire de Sciences de Matériaux et d’Environnement, Faculté des Sciences de SFAX, BP 802, 3018 SFAX, Tunisia

## Abstract

In the title compound, C_9_H_10_N_4_, the phenyl and triazole rings make a dihedral angle of 38.80 (2)°. N—H⋯N hydrogen bonds link the mol­ecules, forming centrosymmetric *R*_2_^2^(8) rings; these rings are inter­connected through a *C*(5) chain, building up a zigzag layer parallel to the (100) plane.

## Related literature

For related literature, see: Altman & Solomost (1993 ▶); Genady & Gabel (2003 ▶); Kanazawa *et al.* (1988 ▶); Karanik *et al.* (2003 ▶); Hashimoto *et al.* (1990 ▶); Allouch *et al.* (2004 ▶). For a discussion of hydrogen-bond patterns, see: Bernstein *et al.* (1995 ▶); Etter *et al.* (1990 ▶).
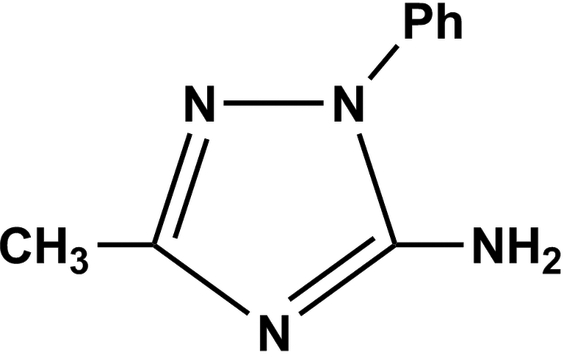


## Experimental

### 

#### Crystal data

C_9_H_10_N_4_
*M* *_r_* = 174.21
Monoclinic, 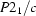

*a* = 8.5110 (5) Å
*b* = 11.2490 (8) Å
*c* = 10.1048 (7) Å
β = 101.866 (4)°
*V* = 946.76 (11) Å^3^
*Z* = 4
Mo *K*α radiation
μ = 0.08 mm^−1^
*T* = 296 (7) K
0.49 × 0.14 × 0.08 mm

#### Data collection

Bruker SMART CCD area-detector diffractometer
Absorption correction: multi-scan (*SADABS*; Bruker, 1998 ▶) *T*_min_ = 0.984, *T*_max_ = 0.997
16028 measured reflections
3882 independent reflections
1997 reflections with *I* > 2σ(*I*)
*R*_int_ = 0.047

#### Refinement

*R*[*F* ^2^ > 2σ(*F* ^2^)] = 0.050
*wR*(*F* ^2^) = 0.148
*S* = 0.94
3882 reflections
124 parameters
3 restraints
H atoms treated by a mixture of independent and constrained refinement
Δρ_max_ = 0.19 e Å^−3^
Δρ_min_ = −0.17 e Å^−3^

### 

Data collection: *SMART* (Bruker, 1998 ▶); cell refinement: *SAINT* (Bruker, 1998 ▶); data reduction: *SAINT*; program(s) used to solve structure: *SHELXS97* (Sheldrick, 2008 ▶); program(s) used to refine structure: *SHELXL97* (Sheldrick, 2008 ▶); molecular graphics: *ORTEPIII* (Burnett & Johnson, 1996 ▶), *ORTEP-3 for Windows* (Farrugia, 1997 ▶) and *PLATON* (Spek, 2003 ▶); software used to prepare material for publication: *WinGX* (Farrugia, 1999 ▶).

## Supplementary Material

Crystal structure: contains datablocks global, I. DOI: 10.1107/S1600536808005801/dn2319sup1.cif

Structure factors: contains datablocks I. DOI: 10.1107/S1600536808005801/dn2319Isup2.hkl

Additional supplementary materials:  crystallographic information; 3D view; checkCIF report

## Figures and Tables

**Table 1:** Hydrogen-bond geometry (Å, °)

*D*—H⋯*A*	*D*—H	H⋯*A*	*D*⋯*A*	*D*—H⋯*A*
N4—H4*A*⋯N3^i^	0.886 (8)	2.110 (8)	2.9923 (13)	173.1 (12)
N4—H4*B*⋯N2^ii^	0.917 (8)	2.210 (10)	3.0415 (14)	150.4 (10)

Symmetry codes: (i) 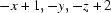; (ii) 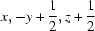.

**Fig. 1.**
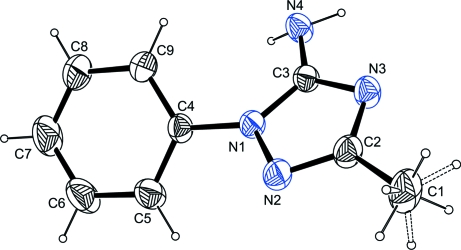
The molecular structure of the title compound with the atom-labelling scheme. Displacement ellipsoids are drawn at the 30% probability level. H atoms are represented as small spheres of arbitrary radii.

**Fig. 2.**
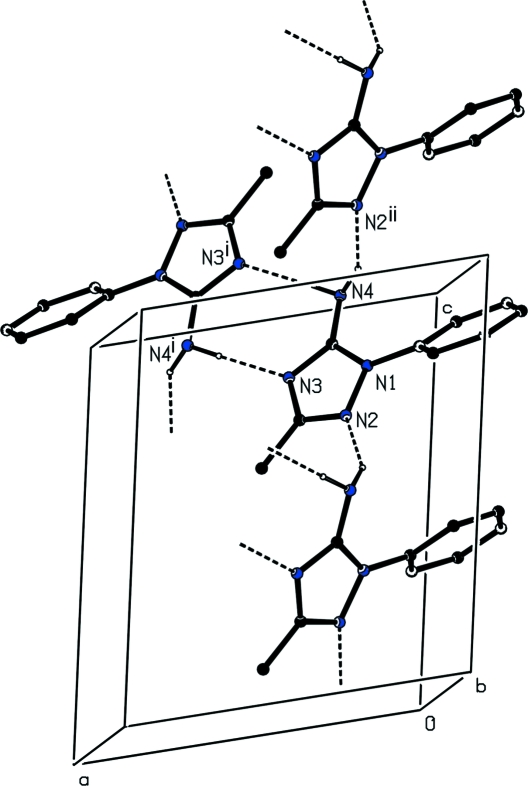
Partial packing view showing the formation of the R22(8) ring and C(5) chains through N—H···N hydrogen bonds drawn as dashed lines. H atoms not involved in hydrogen bondings have been omitted for clarity. [Symmetry codes: (i) -x + 1, -y, -z + 2; (ii) x, -y + 1/2, z + 1/2]

**Fig. 3.**
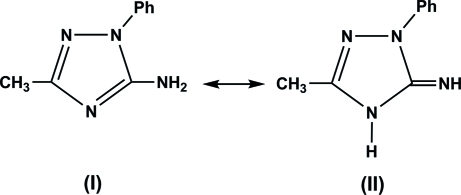
The tautomeric forms of the title compound.

## supplementary crystallographic information

### Comment

The aminotriazoles are crucial heterocyclic substances which have a great interest thanks to their biological and pharmacological activity (Kanazawa *et al.*, 1988; Hashimoto *et al.*, 1990) such as antitumoral and inhibition of cholesterol activity. In addition they have many applications in agriculture domain (Altman *et al.*, 1993). Aminotriazole are useful binucleophilic agents that lead to polycondensed heterocycles (Genady *et al.*, 2003; Karanik *et al.*, 2003). However, the studies that deal with *N^1^-*phenyl-aminotriazole are very limited (Allouch *et al.*, 2004). Until now only a few reactions were reported concerning the addition-cyclizations of bielectrophile compounds with *N*^1^-phenyl-aminotriazoles. In fact, these later are not well identified, this can be explicable by the existence of the tautomer equilibrium. That is why we have undertaken a crystallographic study.

In this paper, we report the synthesis of 5-amino-3-méthyl N^1^-phényl-1,2,4-triazole. The reaction of *N*-phenyl ethyl acetydrazonate with cyanamidegave aminotriazole could lead to structure (I) or its isomer (II) (Scheme). The structure elucidation was achieved by X-ray diffraction, and proved that the reaction occurs cleanly to form 5-amino-3-méthyl N^1^-phényl-1,2,4-triazole (I).

In the title compound, the phenyl and the triazole rings remain planar with mean deviations from planarity of 0.0072 and 0.0049Å respectively. However, the two rings are twisted with respect to each other making a dihedral angle of 38.80 (2)° (Fig. 1).

The occurrence of N—H···N hydrogen bonds links the molecules through inversion centre to form *R*_2_^2^(8) ring (Etter *et al.*, 1990; Bernstein *et al.*, 1995) and these rings are interconnected through C(5) chain to build up a like zigzag layer developping along the (1 0 0) plane (Table 1, Fig. 2)

### Experimental

A mixture containing 3.56 g (0.02 mol) of *N*-phenyl ethyl acetydrazonateand 0.88 g (0.021 mol) of cyanamidein 20 ml of methanol was stirred and heated to reflux for 12 h. The solvent was removed under rotary evaporation. The crude product was washed with ether then recrystallized from methanol to give analytically pure crystals.

### Refinement

All H atoms attached to C atoms were fixed geometrically and treated as riding with C—H = 0.96 Å (methyl) and 0.93 Å (aromatic) with *U*_iso_(H) = 1.2*U*_eq_(Phenyl) or *U*_iso_(H) = 1.5*U*_eq_(methyl). The methyl was found to be statistically disordered over two positions. H atoms attached to nitrogen were located in difference Fourier maps and included in the subsequent refinement using soft restraints (N—H= 0.90 (1)Å and H···H= 1.59 (2) Å) with *U*_iso_(H) = 1.2*U*_eq_(N).

### Crystal data

**Table:**

C_9_H_10_N_4_	*F*_000_ = 368
*M**_r_* = 174.21	*D*_x_ = 1.222 Mg m^−^^3^
Monoclinic, *P*2_1_/*c*	Mo *K*α radiation λ = 0.71073 Å
Hall symbol: -P 2ybc	Cell parameters from 2898 reflections
*a* = 8.5110 (5) Å	θ = 2.5–23.3º
*b* = 11.2490 (8) Å	µ = 0.08 mm^−^^1^
*c* = 10.1048 (7) Å	*T* = 296 (7) K
β = 101.866 (4)º	Prism, colourless
*V* = 946.76 (11) Å^3^	0.49 × 0.14 × 0.08 mm
*Z* = 4	

### Data collection

**Table:**

Bruker SMART CCD area-detector diffractometer	3882 independent reflections
Radiation source: sealed tube	1997 reflections with *I* > 2σ(*I*)
Monochromator: graphite	*R*_int_ = 0.047
*T* = 296(2) K	θ_max_ = 34.4º
φ and ω scans	θ_min_ = 3.0º
Absorption correction: multi-scan(SADABS; Bruker, 1998)	*h* = −11→13
*T*_min_ = 0.984, *T*_max_ = 0.997	*k* = −17→17
16028 measured reflections	*l* = −16→16

### Refinement

**Table:**

Refinement on *F*^2^	Secondary atom site location: difference Fourier map
Least-squares matrix: full	Hydrogen site location: inferred from neighbouring sites
*R*[*F*^2^ > 2σ(*F*^2^)] = 0.050	H atoms treated by a mixture of independent and constrained refinement
*wR*(*F*^2^) = 0.148	*w* = 1/[σ^2^(*F*_o_^2^) + (0.0699*P*)^2^] where *P* = (*F*_o_^2^ + 2*F*_c_^2^)/3
*S* = 0.94	(Δ/σ)_max_ < 0.001
3882 reflections	Δρ_max_ = 0.19 e Å^−^^3^
124 parameters	Δρ_min_ = −0.16 e Å^−^^3^
3 restraints	Extinction correction: none
Primary atom site location: structure-invariant direct methods	

### Special details

**Table:**

Geometry. All e.s.d.'s (except the e.s.d. in the dihedral angle between two l.s. planes) are estimated using the full covariance matrix. The cell e.s.d.'s are taken into account individually in the estimation of e.s.d.'s in distances, angles and torsion angles; correlations between e.s.d.'s in cell parameters are only used when they are defined by crystal symmetry. An approximate (isotropic) treatment of cell e.s.d.'s is used for estimating e.s.d.'s involving l.s. planes.
Refinement. Refinement of *F*^2^ against ALL reflections. The weighted *R*-factor *wR* and goodness of fit *S* are based on *F*^2^, conventional *R*-factors *R* are based on *F*, with *F* set to zero for negative *F*^2^. The threshold expression of *F*^2^ > σ(*F*^2^) is used only for calculating *R*-factors(gt) *etc*. and is not relevant to the choice of reflections for refinement. *R*-factors based on *F*^2^ are statistically about twice as large as those based on *F*, and *R*- factors based on ALL data will be even larger.

### Fractional atomic coordinates and isotropic or equivalent isotropic displacement parameters (Å^2^)

**Table:**

	*x*	*y*	*z*	*U*_iso_*/*U*_eq_	Occ. (<1)
N1	0.22326 (10)	0.20008 (8)	0.83687 (9)	0.0445 (2)	
N2	0.28380 (11)	0.22738 (9)	0.72310 (10)	0.0530 (3)	
N3	0.43302 (11)	0.08529 (9)	0.84645 (9)	0.0500 (3)	
N4	0.29094 (12)	0.06921 (9)	1.02612 (10)	0.0528 (3)	
H4A	0.3772 (12)	0.0290 (10)	1.0662 (12)	0.063*	
H4B	0.2503 (14)	0.1219 (9)	1.0797 (11)	0.063*	
C4	0.08173 (12)	0.25677 (10)	0.85950 (12)	0.0454 (3)	
C3	0.31616 (12)	0.11612 (9)	0.90899 (11)	0.0424 (3)	
C2	0.40669 (14)	0.15538 (11)	0.73467 (12)	0.0535 (3)	
C9	−0.03152 (14)	0.19287 (12)	0.90944 (15)	0.0606 (3)	
H9	−0.0159	0.1124	0.9286	0.073*	
C5	0.05851 (16)	0.37569 (12)	0.82934 (14)	0.0663 (4)	
H5	0.1354	0.4193	0.7968	0.080*	
C1	0.51079 (17)	0.15076 (16)	0.63304 (15)	0.0792 (5)	
H1A	0.5936	0.0925	0.6601	0.119*	0.50
H1B	0.5588	0.2273	0.6270	0.119*	0.50
H1C	0.4471	0.1295	0.5464	0.119*	0.50
H1D	0.4727	0.2070	0.5623	0.119*	0.50
H1E	0.5076	0.0722	0.5953	0.119*	0.50
H1F	0.6192	0.1700	0.6760	0.119*	0.50
C7	−0.19460 (19)	0.36600 (17)	0.89769 (18)	0.0921 (6)	
H7	−0.2890	0.4028	0.9085	0.111*	
C6	−0.0814 (2)	0.42885 (14)	0.84843 (18)	0.0877 (5)	
H6	−0.0991	0.5089	0.8274	0.105*	
C8	−0.16821 (16)	0.24917 (14)	0.93076 (18)	0.0797 (5)	
H8	−0.2428	0.2072	0.9679	0.096*	

### Atomic displacement parameters (Å^2^)

**Table:**

	*U*^11^	*U*^22^	*U*^33^	*U*^12^	*U*^13^	*U*^23^
N1	0.0431 (4)	0.0488 (5)	0.0457 (5)	0.0065 (4)	0.0189 (4)	0.0059 (4)
N2	0.0492 (5)	0.0649 (6)	0.0499 (6)	0.0084 (4)	0.0218 (5)	0.0126 (5)
N3	0.0474 (5)	0.0574 (6)	0.0487 (6)	0.0104 (4)	0.0181 (4)	0.0014 (4)
N4	0.0584 (6)	0.0544 (6)	0.0507 (6)	0.0157 (5)	0.0228 (5)	0.0088 (5)
C4	0.0414 (5)	0.0509 (6)	0.0457 (6)	0.0077 (4)	0.0130 (5)	0.0019 (5)
C3	0.0439 (5)	0.0418 (6)	0.0437 (6)	0.0034 (4)	0.0140 (5)	−0.0003 (5)
C2	0.0480 (6)	0.0669 (7)	0.0500 (7)	0.0048 (5)	0.0200 (5)	0.0064 (6)
C9	0.0476 (6)	0.0612 (8)	0.0777 (9)	0.0036 (5)	0.0243 (6)	0.0070 (7)
C5	0.0711 (8)	0.0611 (8)	0.0754 (10)	0.0174 (6)	0.0353 (7)	0.0164 (7)
C1	0.0679 (8)	0.1133 (12)	0.0673 (10)	0.0214 (8)	0.0392 (7)	0.0151 (8)
C7	0.0709 (9)	0.1057 (13)	0.1108 (14)	0.0396 (9)	0.0445 (9)	0.0214 (10)
C6	0.0926 (11)	0.0767 (10)	0.1056 (13)	0.0420 (8)	0.0481 (10)	0.0290 (9)
C8	0.0534 (8)	0.0923 (11)	0.1032 (12)	0.0100 (7)	0.0385 (8)	0.0146 (9)

### Geometric parameters (Å, °)

**Table:**

N1—C3	1.3459 (14)	C5—C6	1.3811 (18)
N1—N2	1.3874 (11)	C5—H5	0.9300
N1—C4	1.4224 (12)	C1—H1A	0.9600
N2—C2	1.3090 (14)	C1—H1B	0.9600
N3—C3	1.3294 (12)	C1—H1C	0.9600
N3—C2	1.3577 (15)	C1—H1D	0.9600
N4—C3	1.3528 (14)	C1—H1E	0.9600
N4—H4A	0.886 (8)	C1—H1F	0.9600
N4—H4B	0.917 (8)	C7—C8	1.363 (2)
C4—C5	1.3773 (17)	C7—C6	1.369 (2)
C4—C9	1.3785 (15)	C7—H7	0.9300
C2—C1	1.4888 (15)	C6—H6	0.9300
C9—C8	1.3799 (17)	C8—H8	0.9300
C9—H9	0.9300		
			
C3—N1—N2	109.09 (7)	H1A—C1—H1C	109.5
C3—N1—C4	130.56 (8)	H1B—C1—H1C	109.5
N2—N1—C4	120.33 (9)	C2—C1—H1D	109.5
C2—N2—N1	102.42 (9)	H1A—C1—H1D	141.1
C3—N3—C2	103.43 (9)	H1B—C1—H1D	56.3
C3—N4—H4A	109.5 (8)	H1C—C1—H1D	56.3
C3—N4—H4B	114.3 (8)	C2—C1—H1E	109.5
H4A—N4—H4B	115.9 (11)	H1A—C1—H1E	56.3
C5—C4—C9	120.55 (10)	H1B—C1—H1E	141.1
C5—C4—N1	119.18 (9)	H1C—C1—H1E	56.3
C9—C4—N1	120.27 (10)	H1D—C1—H1E	109.5
N3—C3—N1	109.84 (9)	C2—C1—H1F	109.5
N3—C3—N4	125.66 (10)	H1A—C1—H1F	56.3
N1—C3—N4	124.49 (9)	H1B—C1—H1F	56.3
N2—C2—N3	115.20 (9)	H1C—C1—H1F	141.1
N2—C2—C1	122.54 (11)	H1D—C1—H1F	109.5
N3—C2—C1	122.26 (11)	H1E—C1—H1F	109.5
C4—C9—C8	119.55 (13)	C8—C7—C6	119.63 (12)
C4—C9—H9	120.2	C8—C7—H7	120.2
C8—C9—H9	120.2	C6—C7—H7	120.2
C4—C5—C6	118.57 (12)	C7—C6—C5	121.25 (14)
C4—C5—H5	120.7	C7—C6—H6	119.4
C6—C5—H5	120.7	C5—C6—H6	119.4
C2—C1—H1A	109.5	C7—C8—C9	120.39 (13)
C2—C1—H1B	109.5	C7—C8—H8	119.8
H1A—C1—H1B	109.5	C9—C8—H8	119.8
C2—C1—H1C	109.5		

### Hydrogen-bond geometry (Å, °)

**Table:**

*D*—H···*A*	*D*—H	H···*A*	*D*···*A*	*D*—H···*A*
N4—H4A···N3^i^	0.886 (8)	2.110 (8)	2.9923 (13)	173.1 (12)
N4—H4B···N2^ii^	0.917 (8)	2.210 (10)	3.0415 (14)	150.4 (10)

Symmetry codes: (i) −*x*+1, −*y*, −*z*+2; (ii) *x*, −*y*+1/2, *z*+1/2.
